# A Short Description of an Ophthalmia Which Prevailed in Gibraltar and Bombay; with an Account of the Effect of Sea-Water in Arresting Its Progress

**Published:** 1826-04

**Authors:** George Richmond

**Affiliations:** assistant Surgeon of the 4th Light Dragoons.


**Art. III.—*A short Description of an Ophthalmia which prevailed in Gibraltar and Bombay; with an Account of the Effect of Sea-water in arresting its Progress.*** By George Richmond, assistant Surgeon of the 4th Light Dragoons.

Nine years have passed since the first of the following experiments was performed, and found to be of effect in cutting short the progress of an obstinate ophthalmy, which appeared in Gibraltar. The means of practice were so simple and easy, that I wished, long before this time, to have placed them in the pages of some periodical publication; but I was unwilling to form an hypothesis, or advance a remedy, on the proof of one experiment only. For this reason I withheld the result of the practice which I pursued, until the opportunity of more experience would either prove my theory to be unsound, or establish it on the principles of rational induction.

No other instance of the disease, spreading to the extent which I had first seen it, fell under my care before the year 1823, when I happened to be at Bombay: it had there broken out, and grievously affected the children of the charity-schools. This being another well-timed incident for putting my remedies again to the test, I naturally felt very desirous of availing myself of it; and I am happy the result enables me to say, that the disease was arrested in the same decided manner as by that mode of practice which I pursued in Gibraltar.

During the year 1815, while the 2d battalion of the 11th Regiment was quartered at Gibraltar, an ophthalmy broke out, and spread among the men with amazing rapidity, threatening destruction to every eye that came under its influence. The inflammation first made its appearance on the inner edges of the tarsi; thence extended over the surface of the conjunctiva lining the palpebræ. In proportion to the inflammation in this membrane, the meibomian glands became enlarged. In most cases, the intolerance of light was considerable; the margin of the iris was drawn between the chambers like a tense curtain, diminishing the diameter of the pupil, and sometimes giving the pupilary margin the appearance of being sunk, or drawn deeper towards the bottom of the eye than when in health. Light did not contract the compass of the pupil,—not even when the flame of a candle was held before it.

The cornea was put more on the stretch, and had a more glistening appearance, than when in health: in some cases, its augmented convexity was very perceptible. This was owing to the aqueous secretion being greater than the power of absorption. At this stage of the disease, the secretion of tears flowing over the cheeks was copious and hot: in the space of a few hours it was mixed with pus, which could be frequently seen before the matter was discharged from between the eye-lids. By laying hold of the eyelashes, and gently raising up the eyelids, the pus could be seen spread on the lining membrane of the palpebræ, in the form of a patch, about the eighth of an inch in diameter.

The nice adaptation of the globe to its appendages had either, by its rolling motions, squeezed out the serous part of the pus, while that of greater density was retained; or it is not improbable that these patches were the concretions of pus, coagulated by the commixture of tears; for the salt in them may be sufficient to cause coagulation of pus, and by this means alter the consistence and appearance of the discharge.

The vessels and meibomian glands situated on the lining membrane of the palpebræ, rose and swelled above its surface, leaving between them hollow spaces, in which pus was collected in the form and manner I have described. But, when these organs were not in this state, and preserved a smooth uniform swelling over the surface of the membrane, and all the parts were of an equal vascularity, pus was then found spread in an extensive thin layer, alike in thickness, but the consistence much softer and possessed of greater tenuity than that already mentioned. This seems to be the usual character of the pus. It is not improbable that the alteration of membranous structure is the cause of pain, and of the sensation of sand of which the patient complains.

Painful œdematous swellings arose in the integuments round the eye, a shining pale-red colour pervaded the surface of the skin, and an intense shooting pain passed through the eyeball and forehead, accompanied with ardent fever, and a strong hard pulse. These symptoms indicated strong inflammation, and required very active means to check their progress.

The daily admissions into hospital became so numerous, and the cases so severe, as to draw the attention of the military officers of the garrison, to consult and devise with the professional gentlemen the means most likely to arrest and eradicate the disease. A conjecture was then entertained that the current of cold night-air, carried through the barrack windows over the men during sleep, was the cause of the rise and progress of the disease; and, to prevent it from having further effect, orders were given that a number of the windows should be built up half-way, and some completely. But professional opinion preponderated in the belief of the disease having been produced by some noxious quality of the water; and, to prove the correctness of this theory, chemists were employed in analysing the water, which was found to contain no other matter, besides its primary and constituent parts, than a portion of the carbonate of lime; a production naturally to be expected, because the water issues from between beds of that substance.

To prevent the disease from spreading by contact, clean towels were ordered to be distributed occasionally among the men; and, to preserve cleanliness and regularity of conduct, strict regulations were adopted.

All these means, in their application, were very well directed, and were prosecuted with active perseverance; but they had not the most distant effect in restraining the disease. It continued to spread indiscriminately, with unremitted violence, among the men.

The sick-list soon mounted so high as to raise the apprehension of the colonel commanding the regiment, that a representation of the unhealthy state of the men would be forwarded to the Horse Guards, and inevitably prevent the regiment from being called into active service. That ground for such a report should not exist, the most powerful remedies with which I was acquainted were adopted in the process of cure.

The difference of atmospherical temperature during the night from that during the day, was sometimes considerable, but not more than gave health and vigour to the troops, particularly as they were well clothed. Chilly air, wafted over the men during sleep, could not generate ophthalmy to the severity and extent in which it had prevailed. Exposure like this to the night might have brought on other diseases of an inflammatory nature; but how it could produce so much inflammation in the eyes, without extraneous bodies having been carried into them, I could not understand; and, on that account, I concluded this to be but a conjectural inference.

Many of the soldiers were posted on the batteries, and other parts of the garrison, which were plastered with lime; the floors were paved with limestone, which formed a smooth white surface, reflecting the light with great intensity; so that a person with an inactive iris, passing quickly from the shade to this strong light, had a very powerful impression made on the retina.

Frequent reiterations of these changes, in warm climates, bring on amaurosis and glaucoma. Though these sudden changes were not the leading causes of the ophthalmy, yet there was no doubt of their being auxiliaries; for the strong light, falling on the retina in an unusual quantity, had a tendency to excite inflammation.

At the season of the year in which the disease prevailed, the sirocco winds blew with considerable violence, drifting great quantities of sand and dust through the streets of the garrison; especially at Cooperage barracks, where the regiment was quartered. This was owing to the great concourse of people, who met there for the various purposes of commerce, and who had constantly in use many carts and waggons, for the conveyance of merchandise.

The men who were not required to mount guard were employed in blowing and raising rock, to erect and repair public buildings; many also laboured in other departments of government, equally exposed to an atmosphere loaded with clouds of dust, and to the frequent emissions of particles of fire, occasioned by the collisions of the workmen's instruments. The ready and imperceptible admission of these floating particles between the eyelids, will be easily conceived by those who have been much in warm countries; as well as how the friction of particles on the outer coats of the eye produces inflammation through the whole structure.

The causes which I have enumerated appeared to me to have been sufficient to generate and keep up the disease. The means which had been adopted to arrest it had completely failed; and, on that account, a question arose, how did the women and children escape the disease, if the causes were as stated by gentlemen of the profession? The answer was simply this: they were not exposed to the same causes as the men, and to which I have ascribed the disease; and, therefore, I recommended the following experiment as worthy of trial.

That every day, after the men had returned from guard and other public duty, they should rest two hours, in order to cool themselves, and to breakfast. That they should then march to the sea, under the command of a commissioned officer, and bathe for two or three minutes. Every man to carry a vessel with him, and bring home a small quantity of sea-water; and to lie down on his back seven or eight times during the day, while his comrade with one hand opened the eyelids, and with the other dropped a little of the sea-water on the eyeball with a spoon. That every non-commissioned officer should be held responsible for the regularity of these applications. At the same time, I went frequently round the barracks, and explained to the men the propriety of the means, and showed them the method of opening the eyelids, and throwing in the water. I also acquainted them with the great impropriety of any one washing in his comrade's vessel, or using his towel.

These means operated so speedily, as to put a final stop to the career of the disease in the course of eight days. The daily bathing, however, was continued for some months, which proved very beneficial, both in keeping the men cleanly and healthy, so that they soon became as strong as those of any other regiment in the service.

The treatment of the disease consisted of unremitted adherence to the antiphlogistic regimen, and copious abstractions of blood until syncope was produced. In some patients, this diminished action was not necessary; smaller quantities of blood were found sufficient to check the progress of the inflammation: but, in general, it was found necessary to carry bleeding to a considerable length. To act freely on the bowels, and cause counter-irritation, strong cathartics were given; but, in the mean time, care was taken not to excite vomiting, by overloading the stomach with medicines; for, when this was inadvertently brought on, or caused by any spontaneous effort, the fever and inflammatory symptoms ran higher than when vomiting was not induced. But keeping the patient in a constant state of nausea, by small doses of tartar emetic, assisted greatly in subduing the inflammation. To manage this process well, and without exciting vomiting, required the administering hand of the surgeon; for, when it was left to an attendant of the sick, vomiting was occasionally brought on, which tended to frustrate the design. In moderating the circulation, the warm bath was found to be of great service; and, when it brought the patient into a state of faintness, the symptoms of the disease were lessened.

The collyria which I found answer best were solutions of the sulphate of zinc and the sulphate of alum. I made experiments on some others, but found none constringe the vessels, and check the discharge, so readily as these. I give alum, in the acute stage, the preference to zinc.

A weak solution of alum, injected every hour between the eyelids, is the best practice. The surgeon should perform this operation frequently himself, and teach the medical attendants how to do it. In most cases, raising the eyelids gives pain: to avoid this, one or two fingers should be laid on the supercilium, and two of the other hand on the integuments covering the malar process of the cheek-bone; then, by gently pulling the integuments, the eyelids may be separated, while another person throws in the collyrium with a syringe.

It is of great importance to the patient that the surgeon should pay particular attention to this part of practice; for, by washing the purulent matter from the eyes every hour, or according to the violence of the disease, it is prevented from accumulating and fretting the organs. I am certain, by a constant attention to this circumstance, many hundreds of eyes have been saved. By strictly pursuing the line of practice which I have laid down, not one of my patients had his vision in the smallest degree impaired; and it was seldom I detained a patient more than two weeks in hospital, the disease being eradicated within that period.

At Bombay, the Committee of the Society for promoting the

Education of Poor Children have, in the year 1823, and in their eighth annual Report, announced the prevalence of a reiterated ophthalmy among the children of the charity-schools; of which the members state that it was peculiar to the country, and returned periodically. This I beg leave to give in their own words, extracted from the Report:—In page 10th, the members of the committee say, "Notwithstanding, however, for some months past, the children have suffered very considerably from the measles, and from sore eyes; the latter is a disorder which seems peculiar to the country, and to return periodically; it is attended with very acute and painful inflammation, and sometimes occasions a total loss of sight."

The disease spread so rapidly as to lay fifty-five at once in the hospital, and in the course of three weeks half the number belonging to the institution were admitted with it. The progress of the inflammation was equally obstinate and severe as that in Gibraltar; but, the patients being young, and habituated to regularity and temperance, the disease in them was more easily subdued than in the soldiers. But what I had principally to fear was the subtlety of its progress, diffusing its influence to a wide extent. To prevent this, I knew of no remedy so likely to prove effectual in checking its progress, as that which I found so eminently useful in Gibraltar. For that reason, I recommended all the boys in the school to bathe in the sea every day, and a small quantity of sea-water to be thrown in between the eyelids, not less than eight times during the day, in the same manner as I have described for the soldiers; and every other means of the same practice to be used as with them.

By strictly pursuing this scheme, at the end of two weeks the disease had entirely ceased to spread; but, in a considerable number, in whom it had taken a firm root, it required a month to complete the cure.

In comparing the causes, symptoms, and effects of the disease in Bombay, with those of the disease in Gibraltar, they were found to correspond in all their various modifications, except that, by the long reiterated action, the disease in the children had taken a deeper and more stubborn root in the tunics of the eye than in the soldiers'; and could not, therefore, be eradicated by the same means of treatment as those employed for common ophthalmy.

In some patients, an opacity in the cornea had considerably obscured vision; and, although opake matter had been plentifully thrown out between the layers, it had not got time to organize, and was on that account readily removed, and perfect sight restored.

In a great number, chronic vascularity of the conjunctiva, with a suffusion of tears constantly rolling down the cheeks, occurred; and these patients had the diameter of their pupils so much contracted, as to cause imperfect vision. But a few applications of the belladonna broke up the adhesions of the iris, and restored the pupils to their original compass.

In many, on everting the upper eyelids, the conjunctivæ were found covered with small fleshy eminences, which are commonly termed granulations. In the children, they were more crowded towards the angles of the eye than on the tarsal circle, and were soft and pulpy; whereas, in patients advanced in life, they are frequently hard, and difficult to remove.

It was owing to these small bodies that the morbid action and periodical return of the disease were kept up; and it was these causes which also led the members of the committee into the mistake of assigning the disease as the peculiar property of Bombay. In most of these patients, a partial cure or relief had been frequently accomplished; but, these granulations not being removed, the least severity of weather, or exposure of the eyes to dust, occasioned a relapse, and baffled the remedies. However, I found a few applications of the sulphate of copper to these granulations completely remove them, and perfect a cure.

From the time I took charge of these little patients to the period I cut off the disease, and eradicated it from within the walls of the schools, a course of six weeks was occupied; and, although it is now two years since it was arrested, it is not a little gratifying to be able to say that it has not reappeared.

Mr. Morgan, the tutor, informed me that, whenever he observes the least redness in any of the vessels covering the conjunctiva, he has immediate recourse to the sea-water, as a collyrium, with good effect. This washes out any foreign particles which may have been accidentally carried into the eyes, and acts at the same time as a mild collyrium.

